# Practical Observations on the Cure of Wounds and Ulcers on the Legs, without Rest; Illustrated with Cases

**Published:** 1816-09

**Authors:** 


*Practical Observations on the Cure of Wounds and Ulcers on the Legs, without Rest; illustrated with Cases.* By Thomas Whately, Member of the Royal College of Surgeons in London. Second Edition. Octavo, pp. 293. London, 1816.

Accidents and diseases of the lower limbs are among the most common affections which claim the assistance of surgery, and the miseries inflicted by them are frequently great; for they occur principally among the more indigent and laborious classes of the community; who, for the most part, can only purchase recovery by sacrifices more difficult of endurance than even the suffering consequent on disease. We know not a more painful spectacle than that of a poor wretch limping to a day's labour, of fourteen or fifteen hours duration, with legs thickened more from the effects of foul and chronic ulceration, than from the scanty roller which encircles them; or sitting, unoccupied, at home, and consuming, in cataplasms, the loaf destined in better times to form the meal of the hungry groupe by which he is surrounded. Yet these are scenes with which every practical surgeon must be familiar, although we are willing to believe them less frequent of occurrence than at the period of our initiation into the mysteries of physic; when Underwood, Home, Baynton, Bell, and the gentleman, whose work we are about to examine, had not yet enriched with their labours and observations, this too much neglected department of surgery.

Various as are the modes of treatment pursued by different practitioners in wounds and ulcers of the leg (for each has his specific unguent, or plaster, or never-failing balsam), all these varieties may be referred to two grand divisions, according as rest of the diseased limb in the horizontal posture is, or is not, made an essential part of such treatment. The older surgeons, it is well known, invariably prescribed confinement; and many of the moderns still rigidly adhere to this plan. Mr. Whately is a staunch advocate of the opposite system. Compression, of course, is the grand agent by which he operates; but he differs remarkably from Mr. Baynton, as to the mode in which that compression should be applied. Now the fact, in our opinion, stands thus: Sore legs, like many constitutional maladies, admit of being cured by diverse, and often diametrically opposite, means; and he alone is entitled to the character of a consummate surgeon, whose *tact* shall enable him to seize and correctly appreciate the lighter shades of variety and complication, which may decide the preference of one plan of treatment to another; and whose mind is sufficiently vigorous and enlightened to burst through the petty considerations of *nostrum* and *tuum*, and alike avail itself of the resting-chair, adhesive strap, or compress and flannel roller, in the treatment of sore legs, according to the peculiar indication. We have seen Baynton's plan succeed in cases where that of Mr. Whately, long and diligently plied, had before failed; and vice versâ. We have seen rest and emollient poultices effect the *perfect* cure of ulcers upon which compression, variously modified, had exercised a trifling, or only pernicious, influence. It is, however, sufficiently evident, that the cure "without rest," whenever attainable, is always to be preferred; for thus the common avocations of life will suffer little interruption,—an object of no mean importance to patients of the indigent class, affected with ulcers; or to the public institutions of whose inmates they constitute, in general, too large a portion: and every one knows that the cicatrix, formed during a state of rest, frequently gives way, on the restoration of the limb to an erect posture.

Previously to our formal entrance on the business of analysis, we cannot help remarking, that Mr. Whately seems blest with a tolerable share of the good old English spirit of contradiction. No sooner had Sir Everard (then Mr.) Home published to the world his method of treating urethral stricture with caustic, than up starts an antagonist, brandishing his potash bougie, to drive the Scotchman from the stage; and scarcely was the adhesive strap ushered into notice, when its patron found himself assailed by the same vigorous opponent—ambitious to become alike the hero of the bandage and bougie! We seriously recommend Mr. Whately to the " independent electors of Westminster," at the next vacancy in their representation. He will make a famous opposition-man!

Yet the spirit of opposition, we beg leave to observe, in order to secure all those beneficial effects which may result from its operation in science as in politics, should be directed by comprehensive views, blended with candour and liberality; and have, for its exclusive object, the public good rather than private aggrandizement or reward. This admonition, Mr. Whately will do well to bear in mind: and we give it with the less reserve, because he really possesses ardour and talents calculated to exert no despicable influence upon any subject on which he may employ them. To the " rogues and reptiles" who infest the science, we would not deign to proffer our advice.

The first edition of Mr. Whately's book was published sixteen years ago; and, we hope and trust, is well known to most of our readers. On this account, our analysis of its contents will be unusually brief. But we strongly recommend every practitioner, to whom the work is unknown, to peruse the original with attention. He will there find, minutely detailed, a plan of treatment which, experience enables us to assert, will succeed in *certain* cases of wound or ulceration, better than any other.

The *Prefaces* to the first and second editions contain some remarks on the importance of the subject, and superiority of the practice about to be proposed; an attempt to depreciate the value of Mr. Baynton's adhesive strap, by referring its beneficial operation to the bandage commonly employed with it. The benefits of pressure may, it is contended, be more conveniently and effectually obtained by the application of linen compresses and a *flannel* roller. The roller should be of sufficient length and breadth, and always applied by an experienced hand.

Chapter 1.—"Wounds and ulcers on the legs are of more difficult cure than those in other parts of the body." Some, among whom is Mr. Whately, attribute this circumstance to the dependent posture of the limb; others, to the languor of circulation in consequence of its remoteness from the heart. We are disposed to believe, that it is produced by the conjoined operation of both causes; for a wound of the toe is more tardily repaired, *cæteris paribus*, than one occupying the integuments of the tibia or femur. The two principal agents, by which this inconvenience may be remedied, are, the horizontal posture and compression. The latter, when founded on correct indications, is, for the reasons we have before stated, most advantageous: exercise, both from its local and constitutional influence, accelerating the process of reparation, after "good digestion" has been established by the employment of rest and emollient poultices.

Chapter 2.—All wounds and ulcers are either merely local, or connected with some constitutional defect which must be removed ere their cure can be attained. This distinction it is of great importance to remember in practice. All constitutional sores must, in fact, be reduced to a simple state, before their reparation can be radically accomplished; and we can add, that injuries, at first purely local, may, from duration, neglect, or the operation of pernicious causes, ultimately acquire a constitutional character.

Chapter 3.—The horizontal posture and poultices should be prescribed during the inflammation which succeeds "local wounds and other recent accidents." If the inflammation be phlegmonous, evacuants, proportioned to the violence of the symptoms, and spare diet, are indicated: if erysipelatous, tonics and generous food. We should be careful not to lower too suddenly, on the reception of any injury, the diet of those who have previously lived in an intemperate manner: it will be most prudent to indulge, for a time, their vicious habits. The poultice should be renewed twice or thrice a day, if the discharge be copious or offensive. Mr. Whately would not have indulged his quibbling propensities with Mr. Bell, on the operative principle of poultices, had he reflected that they are indebted chiefly for their " emollient relaxing properties," to the " warmth which they contain." Hog's-lard or spermaceti ointment, mean time, may be applied to the surface of the sore. On the establishment of complete digestion, another system is to be pursued. This consists in laying a piece of lint, dipped in oil, or spread with the simplest ointment, to the sore; over this, a pledget of tow covered with calamine or spermaceti cerate; then two or more compresses, made of soft and smooth linen, of unequal size (the smaller one first); and lastly, in applying, over all, a flannel roller. Any inequalities in the surface of the wound may be filled up with different pieces of the lint-plaster; and, in some cases, difficult of cicatrization, it is " extremely serviceable to raise the lint pledgets even *above* the level of the adjoining skin." The wound should be dressed, at first, every twenty-four hours; towards the completion of the cure, every other day. Exercise, or even the most laborious employment may now be freely permitted. Any constitutional debility hostile to the process of reparation, may be remedied by the administration of cinchona, generous diet, and change of air. External means of a more powerful nature may also be occasionally required. When the granulations are foul or flabby, their surface will be advantageously destroyed by nitrate of silver; when exuberant, sulphate of copper is the best application; and hog's-lard should be used as the dressing for some days. Cicatrization may be afterwards accelerated by the employment of preparations of calamine, or weak solutions of the sulphates of zinc or copper. We have found the *vinum opii* an excellent remedy in such cases, especially when the sore has displayed the united characters of torpor and irritability: a combination not unfrequent.

Chapter 4.—Many of the observations contained in the preceding chapter will equally apply to the cure of " local ulcers." Inflammation of the phlegmonous or erysipelatous character, complicated with them, must be subdued by the usual means, previously to the employment of compression. The " foul corroding ulcer," an acute affection, difficult of distinction from the venereal ulcer, will yield to the same plan of repose and cataplasms, combined with opium and tonics internally. Stimulants are not generally to be applied, until ulcers have lost all irritative or inflammatory action. Opium may be given to alleviate severe pain. In some varieties, those especially which exhibit a "black or greenish hue at the bottom," are little inflamed, and of old standing, the early employment of stimulant dressings may be beneficial. The red nitrated quicksilver should be used very sparingly for this purpose. The nitrate of silver is an "excellent remedy;" but more serviceable in small ulcers than in the larger. It should not be too frequently applied. It seldom succeeds, if benefit do not result after using it twice or thrice. The sore should be dressed with mild ointment, when either of these substances has been employed; and, at other times, with a composition of red nitrated quicksilver and lard, in the proportion of ten grains of the former to one ounce of the latter. Callous ulcers may be cured by touching the "root of the callosity" and adjoining parts with nitrate of silver, heaping lint-plaster upon the sore until it rises above the edges, and employing pressure in the ordinary way. To expedite the healing of a sluggish sore, Peruvian balsam may be tried in addition to other topical stimulants, and tonics internally prescribed. Extract of hemlock, given twice a day, sometimes proves very successful in obstinate cases. Should all these fail, some disease of the constitution may be suspected, which will require to be removed, ere the local affection can be successfully treated. Local hindrances to cicatrization may also exist; as diseases of the periosteum, communicating with the ulcer; fistulous sinuses; latent ossification of the aponeurosis of the adjacent muscles; or a "small scirrhus-like body" in the cellular substance. The several remedies in these cases, are, exposure of the periosteum and destruction by caustic; incision of the sinuses, or obliteration by stimulant injections; removal of the ossified portion of membrane; and application of *powerful* caustic to the scirrhous substance. A debilitating plan of diet or medicine will greatly retard the process of reparation. Cutaneous affections, of various character and extent, sometimes attend these ulcers. If the latter be local, the former will generally be so too. They principally arise from the dependent posture of the limb, and compression is essential to their cure; but topical dressings should be conjoined with it, as sulphur ointment, if it be itch: under other circumstances, ointment of tar, of nitrate of quicksilver; or, if a milder remedy be required, simple quicksilver ointment. In all these complaints, the utmost attention should be paid to cleanliness, particularly among the poor.

CHAPTER 5. The constitutional diseases, with which wounds and ulcers on the legs are, for the most part, connected, and which retard their cure, are venereal, scrofulous, and scorbutic. In the *first* case, mercury must, of course, be prescribed; and its influence kept up for some time after every trace of the local malady has disappeared; but we must take care not to treat as syphilitic those "running ulcers" upon which mercury might exert a dangerous or destructive influence: in the *second,* tonics, alteratives, alkalis, exercise, good air; as topical applications, watery sedatives (and particularly a wash made by mixing equal parts of acetate of lead, and sulphate and carbonate of zinc, in water) assisted by compression: For the treatment of the *third,* we are referred to writers on scurvy. Many affections are called scorbutic, although of a nature completely different. Against one variety, occurring about the middle and later periods of life, but not always complicated with ulceration, decoction of elm-bark and the arsenical solution, with the local applications above-mentioned, may be successfully prescribed. There are also two kinds of local ulceration strongly resembling syphilis: the one of an acute character, attended with severe pain, particularly during the night, becoming foul, ichorous, sometimes sloughy and irregular; but spontaneously ceasing to spread, between the twelfth and twentieth day from its commencement, and soon assuming a healthy aspect: the other chronic, consequent on want of cleanliness and pressure, and continuing long in the same state. Besides these, many anomalous affections of the system may occur. Some of them will yield to alterative doses; others to a full course of mercury. Lime-water and decoction of the woods, or hemlock, may be advantageously employed against other varieties.

CHAPTER 6. There is an "erysipelatous inflammation attending wounds and ulcers on the legs" very different from that before alluded to. It is always an acute disease, and usually begins with a cold fit. Its attacks are confined to no habit, age, or season; but are observed more frequently in advanced periods of life, and during cold and wet weather. They, who have taken purgatives or mercurials, seem particularly obnoxious to it. The shivering is succeeded by the usual train of febrile symptoms, and, generally, by sudden developement of cutaneous inflamtion in the morbid limb: yet sometimes there is a considerable interval between the occurrence of the cold fit and inflammation; but the febrile commotion does not subside until the latter has taken place. The affection is sometimes slight; at others, dangerously severe. The inflammation is mostly confined to the leg and foot; but sometimes implicates the whole limb and even the scrotum; it commonly runs its course in from six to fourteen days. It may terminate in resolution, sphacelus, extension of the ulcerative process, or inflammation of the lymphatics, and suppuration of their glands. Sometimes, it is complicated with phlegmon; is then of longer duration, and often followed by a succession of insidious abscesses. Art cannot arrest the progress, although it may moderate the violence, of the disease. In slight cases, little needs be done. When violent, or combined with phlegmon, venæsection, other evacuants, and cooling diet, are indicated: when great debility exists and sphacelus is menaced, opium and tonics. Emollient poultices (with the previous use of warm fomentations) constitute, in either case, the best topical application.

Chapter 7.—A little more quibbling with Mr. Bell on the analogy which subsists between caries of the bones and gangrene of the soft parts. We cannot pause to notice it. "Carious ulcers" are either local or constitutional. In the latter case, our general treatment must be determined by the nature of the malady. But the local plan will be nearly the same in both. Compression and exercise should be employed when all is quiet; for thus the process of exfoliation will be promoted: but during any occasional access of inflammatory symptoms, these should be abandoned, and repose and cataplasms substituted for them. All pieces of exfoliated bone should be removed, as soon as their separation can be ascertained, by manual operation.

Chapter 8.—The cases, in which it might be "dangerous to heal old ulcers on the legs," are not numerous: on the other hand, their cure is, in general, desirable, from the pernicious influence they exert on the constitution. Where, however, from the existence of any "visceral or other complaint," the suppression of discharge from an ulcer might be dangerous, an artificial drain in the arm, or *above* the knee, may be conveniently substituted for it.

Chapter 9.—"The different methods of curing wounds and ulcers on the legs," are here reviewed. The preference unquestionably belongs to that of compression "without rest." But the success of it is frequently marred by negligence in the application of the bandage. The surgeon should always perform this part of the business, himself.

CHAPTER 10.—Surgeons ought not to consider their office at an end, as soon as an ulcer is healed over. The risque of relapse is often great. To prevent this, a roller should be kept on in recent cases, for several months; in those of a more inveterate nature, for years; and re-applied every morning. The cicatrix also should be now and then inspected; all foulness obviated by occasional ablution, not soaking or immersion; and scaly eruptions may be removed by the *sparing* use of any of the unguents mentioned in the fourth chapter.

We now come to "*cases of local ulcers on the legs.*" They are one hundred and sixty-seven in number; and occupy no less than one hundred and fourteen pages of a goodly octavo. What wicked publisher could have put it in the head of Mr. Whately thus unnecessarily to swell the size of his volume? or what possible good can result to the *science* from the narration of such cases as the following, we are utterly at a loss to comprehend!

Case CXLVIII.—Jane Davis, aged forty-five, No. 10, Coppice Row, Spa Fields, applied in 1798, for the cure of two very painful ulcers on the left leg, of three months standing. She was cured in six weeks."

Now, we will take upon us to say, that, if Mr. Whately had simply proclaimed the cure of fifty dozen of sore legs, instead of thirteen, no one acquainted with his character, would have called his veracity in question; and, with great economy of trouble to himself and of expence to the purchaser, every useful purpose would have been fully answered. Indeed, had we been strangers to the honourable character of Mr. Whately, and restricted our inspection to the "catalogue of cures," we should not have hesitated to denounce the author as a quack, and his work as an unwieldy advertisement—the Daniel Lambert of its species. But if our author's *zeal for science must* be gratified by the exhibition of the cases, why did he not throw them into the form of tables? Thus arranged, they would not have occupied the fourth of a sheet; and by a little compression of style, and the employment of a modest type, the whole work might have been reduced within a third of its present limits; and, like a dropsical limb, acquired in vigour and utility, what it lost in bulk. We are always sorry to see that which is really good and useful rendered needlessly expensive.

The *Appendix* contains some additional remarks on the structure and application of bandages, intended for the instruction of the " younger part of the profession," and an antidote to the opinions of Mr. Baynton respecting the inconveniences of the flannel roller and superiority of the adhesive strap. On this point, our sentiments have been already delivered.

In the *Postscript,* it is observed, that moderate exercise may be allowed with advantage in those cases of ulcer not yet in a state to admit of " full pressure and laborious exertion, provided the limb be supported by the gentle compression of a bandage applied over the poultice. Mr. Whately prefers a poultice made of stale *white* bread, *milk,* and hog's lard or sallad oil. Now, why white bread is better than brown for this purpose, or milk preferable to water, we are not cunning enough to conjecture. This circumstance would not have been worth mentioning, but that we have seen the scarcity of white bread and milk become the source of much vexation and expence to the poor, when they have had a cataplasm to prepare for some sick or maimed relative: and we are confident, that the milk, at least, will be better employed in " comforting the bowels" of the patient, than in plastering his outside.

We cannot close this volume without severely reprobating the fulsome language in which the dedication is couched. Dr. Ruddiman, every one knows, is a highly respectable and meritorious physician; and we truly commiserate the painful situation in which the officious zeal of mistaken friendship has placed him. For extravagant compliment, however it may gratify the coarse appetite of " belles and boobies," is to the high and delicate mind the grossest insult that can be offered,—the most deadly satire that can be levelled against it.

---

